# How well does pre-service education prepare midwives for practice: competence assessment of midwifery students at the point of graduation in Ethiopia

**DOI:** 10.1186/s12909-015-0410-6

**Published:** 2015-08-14

**Authors:** Tegbar Yigzaw, Firew Ayalew, Young-Mi Kim, Mintwab Gelagay, Daniel Dejene, Hannah Gibson, Aster Teshome, Jacqueline Broerse, Jelle Stekelenburg

**Affiliations:** 1 Jhpiego, Addis Ababa, Ethiopia; 2 Jhpiego, Paris, France; 3 Ministry of Health, Addis Ababa, Ethiopia; 4 Vrije Universiteit, Amsterdam, Netherlands; 5 Medical Centre Leeuwarden, Leeuwarden, Netherlands

## Abstract

**Background:**

Midwifery support and care led by midwives is the most appropriate strategy to improve maternal and newborn health. The Government of Ethiopia has recently improved the availability of midwives by scaling up pre-service education. However, the extent to which graduating students acquire core competencies for safe and effective practice is not known. The purpose of this study was to evaluate the quality of midwifery education by assessing the competence of graduating midwifery students.

**Methods:**

We conducted a cross-sectional study to assess the competence of students who completed basic midwifery education in Ethiopia in 2013. We interviewed students to obtain their perceptions of the sufficiency and quality of teachers and educational resources and processes. We assessed achievement of essential midwifery competencies through direct observation, using a 10-station Objective Structured Clinical Examination (OSCE). We calculated average percentage scores of performance for each station and an average summary score for all stations. Chi-square test, independent sample *t* test, and linear regression analysis were used to assess the statistical significance of differences and associations.

**Results:**

We assessed 484 graduating students from 25 public training institutions. Majority of students rated the learning environment unfavorably on 8 out of 10 questions. Only 32 % of students managed 20 or more births during training, and just 6 % managed 40 or more births. Students’ overall average competence score was 51.8 %; scores ranged from 32.2 % for manual vacuum aspiration to 69.4 % for active management of the third stage of labor. Male gender, reporting sufficient clinical experience, and managing greater numbers of births during training were significant predictors of higher competence scores.

**Conclusions:**

The quality of pre-service midwifery education needs to be improved, including strengthening of the learning environment and quality assurance systems. In-service training and mentoring to fill competence gaps of new graduates is also essential.

## Background

Ethiopia has made impressive progress towards Millennium Development Goal 4: it is one of seven high mortality countries that have reduced under-five mortality by two-thirds or more before the 2015 deadline [1]. However, absolute levels of maternal and newborn mortality remain high. The 2011 Ethiopia Demographic and Health Survey reported a maternal mortality ratio (MMR) of 676 deaths per 100,000 live births and a neonatal mortality rate (NMR) of 37 deaths per 1000 live births [2].

Now more than ever there is a global consensus regarding the contribution of midwifery care and midwives to reduce maternal and newborn mortality. Competent midwives can provide 87 % of essential care needed for women and newborns and universal coverage with quality midwifery care can prevent more than 80 % of maternal and newborn deaths [3–5].

However, skilled care coverage in Ethiopia is low with only 10 % of births assisted by a doctor, nurse, or midwife [2], which is extremely low even by the standards of sub-Saharan Africa [6]. A shortage of midwives has been an important bottleneck to increasing skilled midwifery care and, consequently, to improving maternal and newborn health outcomes in Ethiopia. In fact, the midwifery workforce including midwives is estimated to meet only 32 % of the need for maternal and newborn health care in Ethiopia, leaving the needs of the vast majority of women and their children unaddressed [5].

Ethiopia had only 0.3 doctors, nurses and midwives per 1000 population when the World Health Organization identified a workforce threshold of 2.3 per 1000 population to achieve high coverage with essential interventions, including those necessary to meet health millennium development goals in 2006 [7]. Since then the Government of Ethiopia has invested in pre-service education to increase availability of the midwifery workforce. It expanded direct entry diploma and degree programs and in 2011 launched an accelerated midwifery training initiative that provided a one-year post-basic training program for diploma-level nurses. As a result of the rapid scale-up of pre-service education, the number of midwives in Ethiopia has increased markedly in recent years, rising from 1275 in 2008 [8] to 6925 in 2014 [5]. But availability of midwives may not translate into improved maternal and newborn health outcomes unless we ensure midwives master the essential knowledge, skills, and attitudes during their pre-service education [3]. This is potentially a challenge in Ethiopia, given high student enrollment, a shortage of qualified faculty, resource constraints, and low caseloads and questionable quality of care at clinical training sites [9–11].

The objective of this study is to generate evidence on the quality of midwifery education by assessing competence of students at the point of graduation. Research on the competence of graduating midwifery students contributes to the much needed evidence base on midwifery workforce development in Africa. It is particularly important for local training institutions to review and improve the quality of pre-service education. The Ethiopian Ministry of Health and Midwifery Association can also use it to identify priority health workforce strengthening strategies.

## Methods

### Study design and sample

This paper reports baseline information on the competence of midwifery students at the point of graduation from public training institutions. It is part of a larger evaluation study that will employ a pre-post design to assess changes in competence levels as a result of technical assistance by the USAID-funded Strengthening Human Resources for Health Project to increase availability of competent midwives. At the time of this baseline study, 20 public universities and 22 public TVET (technical and vocational education and training) colleges offered pre-service education for midwives. All 22 TVET colleges and 8 (out of 20) universities had a graduating class in 2013. Eight universities and 17 TVET colleges were included in this study. Five TVET colleges were excluded because of inaccessibility, inadequate information on graduation status or decision to shut down the program.

A total of 2340 midwifery students (1988 from TVET colleges and 352 from universities) were expected to graduate from these 25 training institutions in 2013. Separate representative samples were calculated for university (185) and TVET (326) programs with assumptions of 95 % level of confidence, 80 % statistical power, 44 % competence level (adopted from a 2008 national emergency obstetric and newborn care survey [11] that reported knowledge score of midwives in maternal care and we selected the score that yielded the largest sample size), 10 % expected increment, and design effect of 1. We randomly selected 20 students per TVET college and 24 students per university from the registrar lists to participate in the study. Of these 532 students, 484 students (336 from TVET colleges and 148 from universities) participated in the study. The average number of participants at each institution was 18.5 (range: 7–24) for universities and 19.7 (range: 18–20) for TVET colleges. The main reasons for relatively lower participation from universities were the inability to recall students after they completed their exams and the overlap of the data collection schedule with student preparations for graduation.

### Data collection

Data were collected in June/July 2013 from students who completed their education through interview and direct observation of performance. Data collectors interviewed students privately to obtain information on their background characteristics and perceptions of whether instructors, resources, and infrastructure were adequate and effective using a three-point scale (yes, partially, no). Students were also asked to report how many births they attended during training.

After the interview, the competence of students was assessed using an Objective Structured Clinical Examination (OSCE), a testing format widely believed to generate valid and reliable conclusions [12–15]. The content of the OSCE was drawn from the essential competencies for basic midwifery practice defined by the International Confederation of Midwives (ICM), which serve as expected outcomes of pre-service education [16]. Three additional competencies (3^rd^, 5^th^ and 10^th^ tasks below) were tested based on national needs. We mapped out 10 OSCE stations, or tasks, from these competencies. Each task consisted of 5 to 13 skills steps. The 10 OSCE stations were: 1) assisting normal delivery, 2) active management of the third stage of labor, 3) vacuum-assisted delivery, 4) history taking in providing focused antenatal care, 5) manual vacuum aspiration, 6) newborn resuscitation, 7) partograph interpretation, 8) postpartum counseling, 9) applying medical eligibility criteria for family planning provision, and 10) integrated management of childhood illness (IMCI).

At each manned OSCE station, the assessor explained the assessment process to the student, provided a case scenario, and asked the student to perform the task, either with a mannequin and/or a simulated patient. The assessor observed and noted whether the student satisfactorily performed each step in the observation checklist for that OSCE station. Each student rotated through all 10 OSCE stations and spent 10 min at each station. Senior midwives with experience in performance assessment administered the assessment. Prior to deployment, they attended a five day training, which covered conducting the OSCE, interviewing skill, obtaining informed consent, maintaining confidentiality, and pre-testing of instruments. Assessors validated the content and tools of the OSCE thoroughly during the training. We ensured midwifery instructors were not assigned to their own training institutions and supervisors closely monitored the data collection process.

### Data analysis

We used the Census and Survey Processing System Program (CSPro Version 5.0) for data entry and SPSS Version 20 for statistical analysis including descriptive (proportion, mean, range) and analytic statistics (Chi-square test, independent sample *t*-test, linear regression). For the competence assessment, we performed reliability scale analysis to examine the consistency or redundancy of items within each task or station. The internal reliability of items was found to be acceptable (ranging from 0.61 for the family planning station to 0.86 for the newborn resuscitation station), with 7 of the 10 stations having Cronbach alpha values of 0.7 or more.

To evaluate student perceptions of the learning environment, we calculated the percentage of students who responded “yes” to questions about the adequacy and effectiveness of the educational resources and instructors at their training institution and clinical practicum sites. We also calculated the percentage of students who met minimum national and global standards [17] for the number of births attended while in training (20 and 40 births, respectively).

To measure level of competence, we calculated the percentage of steps that students completed satisfactorily at each OSCE station. Mean scores for each station were averaged to create a summary score for overall competence; each of the 10 tasks or OSCE stations contributed equally to this summary score. To judge whether students would be considered competent by national standards, we also calculated the proportion of study participants who scored 60 % or higher- the passing mark for professional courses in Ethiopian higher education institutions.

The Chi-square test was used to assess difference in gender distribution of students between university and TVET programs as well as difference in meeting national standards for number of births assisted during training by type of educational program. We applied independent sample *t*-test to assess difference in competence scores across gender and type of education programs. Bivariate and multivariate linear regression analyses were used to identify factors associated with achievement of competence. These included student’s gender and age; type of educational program; student perceptions of resources and learning in the classroom, the skills lab, and clinical practicum sites; and the number of births attended during training. We computed coefficients with 95 % confidence interval (CI). Independent variables with p-values less than 0.3 in the bivariate analysis were selected for multivariate linear regression. A p-value of less than 0.05 was considered statically significant. We checked statistical modeling assumptions (outliers, linearity, normality, homoscedasticity, and multicollinearity of the data) before carrying out statistical tests, and we found no violations.

### Ethical considerations

The study was approved by the Johns Hopkins School of Public Health Institutional Review Board. We obtained oral informed consent from participating students and permission from deans of the training institutions before collecting data.

## Results

### Description of study participants

A total of 484 students graduating from 25 pre-service midwifery education institutions participated in the study, yielding an overall response rate of 91 %; the response rate was 98.8 % for technical and vocational education and training (TVET) and 77.1 % for university students. Of these, 217 (44.8 %) were graduating from direct entry TVET program, 119 (24.6 %) from post-basic TVET program,, and 148 (30.6 %) from university program. Females accounted for 64.9 % of all study participants; females were more likely to be enrolled in TVET (74.5 %) than university program (42.6 %) (Pearson Chi-square = 46.56, degree of freedom = 1, *p* < 0.001). The mean age of study participants was 21.7 years, and the youngest and the oldest were 18 and 30 years, respectively. Overview of midwifery education programs in Ethiopia is provided in Table 1.

**Table 1:** Overview of basic midwifery education programs in Ethiopia

Type of education program	Qualification	Duration	Curriculum	Entry requirement
Direct Entry TVET (Technical and Vocational Education and Training) program	Diploma	3 years	Competency-based curriculum	Successful completion of 10 years of education plus fulfilling entrance requirement for TVET set by the Ministry of Education
Post-basic TVET (Technical and Vocational Education and Training) program	Diploma	1 year	Competency-based curriculum	Successful completion of 3 years of diploma nursing education
University program	Degree	4 years	Subject-based curriculum	Successful completion of 12 years of education plus fulfilling entrance requirement for higher education set by the Ministry of Education

### Perceptions of study participants about the learning environment

On 8 of 10 questions, most students rated the learning environment negatively. Only 44.6 % of students felt skills lab assistants were effective in supporting students, and 28.9 % thought their number was adequate. Clinical preceptors received the lowest ratings: only one in five students said they were adequate (19.2 %) and available to support students at practicum sites (21.5 %). Students rated the availability and helpfulness of resources more highly in the classroom than the skills lab (43.8 and 28.3 %, respectively). Classroom instructors received a relatively more favorable assessment: over half of students thought classroom instructors were effective (56.2 %) and fair (67.4 %). University students were less likely than TVET students to be satisfied with the learning environment (*p* < 0.001). However, perceptions of the learning environment did not significantly differ between direct entry and post-basic TVET students, except for classroom resources and the adequacy of preceptors at practicum sites (Table 2).

**Table 2:** Percent of study participants who responded positively to questions on the learning environment, by type of education program

Perceptions	TVET programs			University programs (*n* = 148)	All programs (*n* = 484)	*P*-value	
	Post-basic (*n* = 119)	Direct entry (*n* = 217)	All TVET (*n* = 336)			TVET post-basic versus direct entry	All TVET versus university
Classroom resources and learning							
Classroom learning resources were available and helpful	60.5	49.8	53.6	21.6	43.8	0.038	<0.001
Number of instructors was adequate	55.5	51.2	52.7	20.9	43.0	0.260	<0.001
Instructors were effective in facilitating learning	64.7	68.2	67.0	31.8	56.2	0.297	<0.001
Instructors were fair and unbiased in assessing learning	79.8	74.2	76.2	47.3	67.4	0.152	<0.001
Skills learning lab resources and learning							
Skills lab resources were available and helpful	38.7	37.3	37.8	6.8	28.3	0.450	<0.001
Number of skills lab assistants was adequate	33.6	41.0	38.4	7.4	28.9	0.112	<0.001
Skill lab assistants were effective in supporting students	52.9	59.4	57.1	16.2	44.6	0.150	<0.001
Clinical resources and learning							
Number of preceptors in practicum sites was adequate	36.1	22.1	27.1	8.8	21.5	0.004	<0.001
Clinical teachers and preceptors were available during the scheduled time and supported students	28.6	20.7	23.5	9.5	19.2	0.070	<0.001
Practical experience was sufficient to master midwifery competencies	54.6	61.8	59.2	27.7	49.6	0.124	<0.001

Note: Students responded to questions using a three-point scale: yes, partially, and no. Only “yes” responses are reported here

**Fig. 1:**
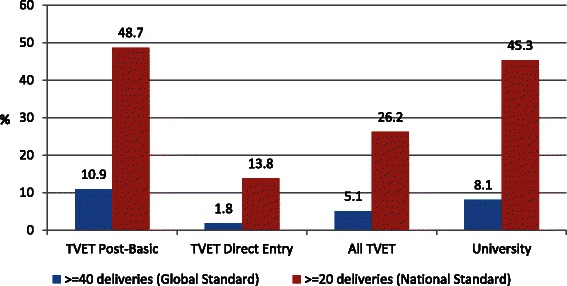
Percent of study participants who met minimum national and global requirements for the number of births attended during midwifery education

Only 32 % of all students had attended 20 or more births (a national standard) and a much smaller 6 % had attended 40 or more births under supervision. University students were almost twice as likely as TVET students to meet the national standard of assisting at least 20 births (45.3 and 26.2 %, respectively, Pearson chi-square =17.18, degree of freedom = 1, *p* < 0.001) (Fig. 1). Likewise, post-basic TVET students were more than three times as likely as direct entry TVET students to meet the national standard (48.4 and 13.8 %, respectively, Pearson chi-square = 48.5, degree of freedom = 1, *p* < 0.001). The median number of births attended by study participants was 11; and 20 students (4.1 %) did not report attending even a single delivery during training.

### Achievement of core competencies

**Fig. 2:**
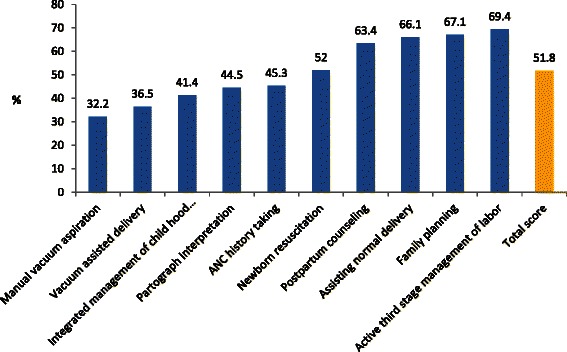
Mean performance scores at each OSCE station and overall mean performance score for all study participants

Student performance varied widely across OSCE stations (Fig. 2). Students performed relatively well in active management of the third stage of labor (69.4 %), clinical decision-making skills in family planning service provision (67.1 %), assisting normal delivery (66.1 %), and postpartum counseling (63.4 %). The lowest performance was recorded for manual vacuum aspiration (32.2 %) and vacuum assisted delivery (36.5 %) tasks. The summary score reflecting average performance across all ten OSCE stations was 51.8 %. Moreover, only 31.6 % of students had an overall performance score that was equal to or greater than the national passing standard of 60 %.

Gender and type of educational program appear to influence achievement of specific competencies. Male students scored significantly higher than their female counterparts in majority of the stations; namely, family planning, antenatal care, partograph, vacuum assisted delivery, manual vacuum aspiration, and integrated management of childhood illness. University students significantly outperformed TVET students in five OSCE stations: vacuum assisted delivery, manual vacuum aspiration, partograph interpretation, family planning, and integrated management of childhood illness. The converse was true for three stations: assisting normal delivery, active management of the third stage of labor, and newborn resuscitation. In the context of TVET colleges, students in direct entry programs performed significantly better than students in post-basic programs in three stations (vacuum assisted delivery, newborn resuscitation, and partograph interpretation), while post-basic students outperformed direct entry students in one station (integrated management of childhood illness) (Table 3).

**Table 3:** Mean performance score at each OSCE station, by gender and education program

OSCE station	Gender			Training program					
	Male (*n* = 170)	Female (*n* = 314)	*P*-value*	TVET program			University program (*n* = 148)	*P*-value*	
				Post-basic (*n* = 119)	Direct entry (*n* = 217)	ALL TVET (*n* = 336)		TVET post-basic versus direct entry	All TVET versus university
Assisting normal delivery	65.6	66.4	0.715	67.8	72.6	70.9	55.3	0.073	<0.001
Active management of 3^rd^ stage of labor	69.4	69.3	0.968	72.9	71.0	71.7	64.1	0.392	<0.001
Vacuum assisted delivery	44.9	31.8	<0.001	32.2	35.8	34.5	40.9	0.269	0.016
ANC history taking	50.4	42.6	0.001	45.0	46.6	46.0	43.8	0.579	0.344
Manual vacuum aspiration	35.5	30.4	0.048	25.5	32.4	29.9	37.4	0.015	0.007
Newborn resuscitation	53.6	51.2	0.424	50.0	58.1	55.2	44.8	0.020	0.001
Partograph interpretation	50.0	41.6	0.003	33.4	41.1	38.4	58.4	0.022	<0.001
Postpartum counseling	64.2	63.0	0.570	65.7	63.9	64.5	61.0	0.412	0.092
Applying medical eligibility criteria in family planning provision	71.8	64.5	0.005	61.8	66.6	64.9	71.9	0.151	0.014
Integrated management of childhood illness	49.5	36.9	<0.001	43.6	37.5	39.7	45.2	0.038	0.024

*P-value computed using independent sample *t*-test

### Factors associated with achievement of core competencies

Bivariate linear regression analysis found that five factors were significantly and positively associated with student performance on the OSCE: male gender, older age, attending more births during training, perceived availability of skills lab resources, and perceived sufficiency of clinical learning experience. The type of educational program and other elements of the learning environment were not associated with student competence (Table 4).

**Table 4:** Bivariate and multivariate linear regression analysis of factors associated with competence score of study participants

Variable	Bivariate linear regression		Multivariate linear regression	
	Coefficient (95 % CI)	*P*-value	Coefficient (95 % CI)	*P*-value
Male gender	5.707 (2.887, 8.526)	<0.001	5.429 (2.517, 8.341)	<0.001
Age (in years)	0.988 (0.297, 1.679)	0.005	0.460 (−0.241, 1.160)	0.198
University educational program	0.716 (−2.252, 3.684)	0.636	-	
Number of births managed during training	0.183 (0.074, 0.291)	0.001	0.164 (0.055, 0.273)	0.003
Felt classroom resources were available and helpful	−0.131 (−2.888, 2.626)	0.926	-	
Felt number of instructors was adequate	2.191 (−0.565, 4.947)	0.119	2.428 (−0.529, 5.385)	0.107
Felt instructors were effective in facilitating learning	−2.469 (−5.217, 0.278)	0.078	−3.543 (−6.360, −0.726)	0.014
Felt instructors were fair and unbiased in assessing	−2.742 (−5.648, 0.164)	0.064	−2.794 (−5.710, 0.122)	0.060
Felt skills lab resources were available and helpful	3.593 (0.574, 6.612)	0.020	2.749 (−0.528, 6.025)	0.100
Felt number of skills lab assistants was adequate	2.835 (−0.171, 5.840)	0.064	2.239 (−1.043, 5.520)	0.181
Felt skills lab assistants supported students effectively	0.073 (−2.678, 2.824)	0.959	-	
Felt number of preceptors was adequate	2.070 (−1.254, 5.395)	0.222	0.204 (−3.92, 4.321)	0.922
Felt clinical teachers and preceptors supported students	1.892 (−1.575, 5.359)	0.284	0.636 (−3.651, 4.923)	0.771
Felt clinical experience was sufficient	4.619 (1.915, 7.323)	0.001	4.650 (1.863, 7.437)	0.001

Three of these five factors remained significant in the multivariate analysis: gender, number of attended births, and sufficient clinical learning experience. Instructors’ effectiveness in facilitating learning also proved to be significant (though negatively) in the multivariate model. The two strongest factors were gender and clinical experience. The average performance score of male students was higher than their female counterparts by 5.429 points (95 % CI = 2.517, 8.341; *p* < 0.001), after controlling for the effects of other variables. The average score of students who said they had enough clinical experience to master midwifery competencies was higher by 4.65 points (95 % CI = 1.863, 7.437, *P* = 0.001). For each additional birth attended, students’ average performance score increased by 0.164 points (95 % CI = 0.055, 0.273; *p* = 0.003). Surprisingly, students who said classroom instructors were effective in facilitating learning had lower scores, by a factor of 3.543 (95 % CI = −6.360, −0.726; *p* = 0.014).

## Discussion

Midwifery support and care particularly one led my midwives working in the context of collaborative interdisciplinary team and integrated health system can improve health outcomes for women and infants and be very cost-effective. However, the effectiveness of midwives to do so at least partly depends on the quality of their educational preparation [3–5]. This study sought to verify the quality of midwifery education by assessing competence of midwifery students at graduation level against the ICM essential competencies for basic midwifery practice [16] and local health service needs. We found that the mean performance score was unsatisfactory and that most midwifery students at public training institutions in Ethiopia did not master the essential competencies for safe and effective practice. This raises important concerns regarding the quality of pre-service education. Performance scores were lowest for basic emergency obstetric skills of vacuum assisted delivery and manual vacuum aspiration, notwithstanding the importance of prolonged/obstructed labor and unsafe abortion as leading causes of maternal death in Ethiopia [18]. Our findings are similar to the few published studies assessing the competence of newly qualified midwives and nurses. In Afghanistan, a study evaluated six core competencies of midwives 2.6 years after they were deployed to their workplaces by observing their performance with anatomical models. Midwives working at rural clinics scored 63.2 % and those at hospitals scored 57.3 %, suggesting that pre-service education in Afghanistan did not fully prepare students [19]. A small study of newly qualified registered nurses at two South African hospitals also reported that recently graduated nurses were not competent, with an average performance score of 34.05 % [20]. In the United Kingdom (UK), a longitudinal qualitative study that evaluated the preparedness of newly qualified midwives to provide clinical care found that newly qualified midwives lacked confidence in key areas and suggested the presence of gaps in the curriculum [21].

Our findings on the learning environment, while subjective, confirm widespread problems with quality and adequacy of teachers, educational resources, and the teaching and learning process, which are essential for quality pre-service education [22–27]. The practical learning experience was particularly deficient, typified by the small number of births managed by students. In this study, each midwifery student managed 11 births, on average, compared with the national standard of 20 births and the global standard of 40 births [17]. This is important because we also found that students who reported sufficient clinical experience and assisted greater number of births had higher competence scores. However, the availability of teachers and educational resources as well as perceived effectiveness of teachers’ support for practical learning was not associated with student competence in this study. Even more surprising, students who perceived their classroom teachers to be effective in facilitating learning scored lower on the OSCE, warranting further exploration. One possible explanation is that students who rate classroom teachers favorably may do so because they are more comfortable with theoretical learning but less so with practical learning and competence. But these findings must be interpreted cautiously, given the inherent challenges of interpreting self- reported answers.

Our study found that male students achieved a higher competence than their female counterparts. This contradicts research reports from around the world. An integrative literature review by Johnson and colleagues [22] concluded that gender did not contribute to academic performance. In contrast, a series of studies that have investigated the effect of gender on the academic success of nursing students [28], clinical performance of medical students [29, 30], and clinical knowledge [31] and communication and interpersonal skills [32] of examinees in the United States Medical Licensure Examination have all concluded that women are better performers. The inferior performance of women in our study perhaps reveals the inadequacies of affirmative action program in the Ethiopian education system introduced to redress the considerable gender gap. Although affirmative action provides female students preferential admission to tertiary educational programs with lower grades [33], our finding raises questions about the availability and adequacy of academic support to help them succeed once they are enrolled.

Even though there was no difference in the overall competence between university and TVET students, TVET students outperformed on some tasks, while university students outperformed on others. This suggests that the two programs have different strengths and weaknesses. While there are no contemporary studies comparing vocational and university education programs [22], our finding is in agreement with a study from the UK that compared nurses prepared through diploma and degree programs. The UK study found little difference in overall and specific competencies between the two groups, based on self- and manager ratings [34].

### Implications

There is no doubt that midwifery services and midwives are crucial to the achievement of national and international goals in reproductive, maternal, newborn and child health, now and beyond 2015 [4]. However, the likelihood that most students were allowed to graduate without ensuring their competence warrants action. The competence, and consequently performance of health workers, has an immediate impact on the quality of health care and population health outcomes [7]. Allowing incompetent midwives to enter the workforce without remediation compromises patient safety, undermines public confidence in the health system, and makes it difficult to meet national goals for improving maternal and newborn health. It also undermines the status of the profession and reduces the self-esteem of practicing midwives, making it harder to recruit, motivate, and retain midwives – thus exacerbating the human resources crisis that the expansion of midwifery education is supposed to address. Hence, our finding begs for action by key players.

It is essential that the Ministry of Health, in collaboration with the Ethiopian Midwifery Association and other partners, take action to strengthen the skills of midwives entering the workforce. An integrative literature review suggests that targeted, repetitive in-service training using effective techniques and conducted in a setting similar to the workplace can improve knowledge and skills and clinical practice behaviors [35]. There is also evidence that supportive supervision coupled with audit and feedback improves health worker competence [7]. If feasible, requiring post-graduation preceptorship can be considered.

Higher education institutions in Ethiopia must strengthen their internal quality assurance systems to ensure the sufficiency and quality of teachers, physical resources, the teaching-learning process, and attainment of essential competencies. Assessment deserves special attention in view of the critical role it plays in driving student learning [36] and ensuring training quality [37]. Students’ low level of competence at graduation indicates the assessment methods in current use do not fully encourage students to master essential competencies and/or help make accurate promotion and graduation decisions. Based on the literature on validity and reliability of assessment [38, 39] as well our own experience, we hypothesize that the sources of the problems in Ethiopia may be lack of attention to formative assessment, under-representation of essential midwifery competencies in the assessment, subjective and less reliable assessment scores, weak performance assessment, and lowering of the pass/fail threshold.

Changing government regulations to subject public higher education institutions to accreditation would ensure their programs are consistent with professional standards [40], encourage institutional improvement and promote appropriate learning environments [41], and improve the quality and relevance of health professionals [42]. Currently, only private higher education institutions in Ethiopia are subject to accreditation [43]. This creates the risk that midwifery education programs at public institutions may not fulfill minimum resource requirements, particularly in the midst of a government push for rapid expansion.

The findings also provide important lessons for the global human resources for health community. Calls for strengthening human resources for health may have succeeded in increasing the quantity of health workers, but guaranteeing the quality of those graduates is harder to achieve. In fact, the intense focus globally on the shortage of health workers may actually have pushed the quality of education lower on governments’ health workforce agenda. World Health Organization guidelines clearly recognize that increasing the number of health workers without ensuring their competence will not strengthen health systems or improve health outcomes [42]. As our study shows, there is a need to refocus attention on the quality of health workers’ education. Countries working to rapidly increase the production of health workers should develop reliable quality assurance systems. Good practices in the successful scale-up of pre-service education that enhance the quality as well as the quantity of health workers should be documented and shared.

### Strengths and limitations

This study is the first national-level assessment of the competence of midwifery students in Ethiopia and one of very few worldwide. It is also notable for the high quality of data on student competence. The assessment tasks are blueprinted from the ICM essential competencies and national priorities for midwifery, increasing the validity of the findings. The OSCE approach provided a reliable measurement of student competence. In addition, the observers were qualified and experienced, and the data collection process was rigorously supervised.

One limitation of the study is that it did not include TVET colleges from two underdeveloped regions (Afar and Somali), due to communication and travel challenges. It is possible that the findings on student competence are slightly higher because of this omission. However, we believe the findings do represent the big picture in Ethiopia, because the study included more than four-fifths of all midwifery training institutions in the country. Another limitation is that performance in a simulated setting may not be the same as performance in a real clinical setting.

## Conclusions

The competence of graduating midwifery students, and hence the quality of pre-service education, was found to be inadequate. Male gender, sufficient clinical experience, and attending more births during training predicted competence, while other variables related to educational inputs and processes, students’ age, and type of educational program were not significant. The inadequate competence level has important implications for the Ministry of Health, training institutions, and regulatory bodies. Effective in-service training, on-the-job mentoring, and supervision are needed immediately to improve the competence of midwifery graduates who are entering the workforce. Without it, the safety of mothers and children will be placed at risk. For the long term, the quality of midwifery education programs at public institutions must be improved through strengthened internal quality assurance systems, external quality checks and accreditation systems.
